# The Haunted House

**Published:** 1863-05

**Authors:** 


### HALL'S JOURNAL OF HEALTH.

Our Legitimate Scope is almost boundless: for whatever begets pleasurable and harmless feelings, promotes Health; and whatever induces disagreeable sensations, engenders Disease.

WE AIM TO SHOW HOW DISEASE MAY BE AVOIDED, AND THAT IT IS BEST, WHEN SICKNESS COMES, TO TAKE NO MEDICINE WITHOUT CONSULTING A PHYSICIAN.

Vol. X.] MAY, 1863. [No. 5.

### THE HAUNTED HOUSE.

WHAT shrinkings within, what an indefinable awe pervades the whole being of a child, at the mention of the ominous words: "The haunted house." It is an appellation more prejudicial to real estate than that of "mad-dog" to a quadruped, or "thief" to human derelicts. There is an old dwelling in Schermerhorn street, Brooklyn, which has a history in this connection. It had a pleasant look about it, and the grounds around it made it a very desirable place of abode, as far as the outside was concerned. Years agone it was put up at auction, and "went for a song" to a stranger, who immediately moved into it. But his health began to give way, he grew thin and pale, broke up house-keeping, and labeled it: "To Let." A New-York merchant chanced to pass that way, and as he had just married a beautiful woman of family and fortune, he concluded, with her approbation, to take the house for a year as an experiment in house-keeping. The handsomest room in the house was occupied as a chamber; as the family-room; in fact, it was the only room which could be conveniently appropriated to that purpose. It was not long before the young wife was observed not to look as well as formerly. She was a woman of a great deal of firmness of purpose, and possessed a high degree of moral courage. Extraordinary noises were heard at night which prevented any sound, refreshing sleep for hours together. At one time fierce sharp cracks would be heard at the head of the bed, against the board; at another, a bureau or closet-door would resound with some heavy thump. A little child would often wake up with the exclamation:

"Mother, this noise won't let me sleep." The servants would leave the house in a body, without explanation; sometimes two sets would come and go within a week. The husband was oblivious to all these things; for he was an active business man, spending the whole day in New-York, and fell asleep almost as soon as his head touched the pillow; scarcely turned over until broad daylight. The wife, being afraid of ridicule, said nothing. She was not conscious of any feeling of fear. "I knew a noise could not hurt me, yet it prevented refreshing sleep." Meanwhile several weeks had passed, when the owner of the house was accidentally met in the street, but so changed in personal appearance, that he was scarcely recognized; the wan, haggard and pale face had fulled out and reddened and become cheery; the gait was confident, the step elastic, and the whole bodily presence was, as it were, reörganized. When congratulated on his altered appearance for the better, he turned it off by some allusion to the cares of house-keeping, change, etc. By this time summer came, and the young wife went to the country, having invited her mother to come and take charge of the household during her absence, giving no instructions, except an apparently casual charge to sleep in her chamber, as it was the finest and most convenient room in the house. On returning in the fall her first observation was, that her mother had been occupying a distant room; but no remark was made in reference to that point for several days, as it was preferred that the mother herself should introduce the subject, but never a word did she utter. When the question was put directly, as to the cause of taking an inferior apartment, the wily matron turned the subject by remarking indifferently that she found she did not sleep very well there, and thought a change to some other part of the house might be advantageous; which was the case.

In a few days two romping young ladies, cousins, from a distant city, came to spend the winter with our heroine, who, in courtesy, gave them the "best" room in the house, the fated chamber. In less than a week they announced their intention of returning home immediately, giving no satisfactory reason for so doing; the hidden cause, however, was divined, and they were transferred to another part of the building, and staid out their full time. Spring came, the year for which the house was taken expired, and it was gladly given up. As there was an indisposition to injure the landlord's property, there was no allusion ever made to the neighbors about the noises; but some one incidentally made the remark one day, that several years before either a murder or a suicide had been committed in that room.

It would be useless to deny that noises were heard in that room; and any one who would say that they were the result of human machinations, would but suggest a greater absurdity than that there were no noises at all. We have heard noises ourselves, when seated around the family fireside; noises as loud and as sudden as the crack of a pistol, within a yard of our elbow. Then, again, there are malign influences in certain localities, in house and field, as impalpable as thin air, and yet as destructive to brain or body as the deadliest agent known to man. Napoleon the First observed that the occupant of a certain sentry-box, for several times in succession, committed suicide; he promptly ordered its total destruction, and a new locality to be selected for the one built in its place; and there were no more suicides. There is many a well, into which, if a man descends, he dies within the hour. See our book on "Sleep," in reference to the selection of localities for the erection of family dwellings.

It is our habit, when persons are on the high horse for narration, to say not a word, ask not a question, until they are evidently "through." Give the narrator a plenty of tether, and he will be very certain to wind himself up in inexplicable contradictions if he is merely romancing. On the other hand, if a true tale is told, it often happens that an incidental remark is dropped, to which the speaker himself attaches but little importance, but which in its connections, upholds to the clear light of day, what otherwise might be painfully intricate, or supernaturally mysterious.

In the course of the above narration, made an hour or more ago in our office, the lady remarked, without the slightest apparent consciousness that it had even a remote bearing on the subject, and, least of all, that it was the key to the unravelment of the mystery: "The sun never shines in that room." No such room can be otherwise than unhealthful, as a human habitation, because there are various gases or emanations, which, with the constantly varying conditions as to dampness, dryness, heat and cold, must have a disturbing influence on the furniture and wood-work. And let it be remarked (without having seen it suggested in print or conversation) that "noises," which the vulgar are so ready to attribute to supernatural influences, are never heard as issuing from a solid blind-wall, where there is no wood-work whatever.

